# Thermodynamics of protein denaturation at temperatures over 100 °C: CutA1 mutant proteins substituted with hydrophobic and charged residues

**DOI:** 10.1038/srep15545

**Published:** 2015-10-26

**Authors:** Yoshinori Matsuura, Michiyo Takehira, Yasumasa Joti, Kyoko Ogasahara, Tomoyuki Tanaka, Naoko Ono, Naoki Kunishima, Katsuhide Yutani

**Affiliations:** 1 RIKEN SPring-8 Center, 1-1-1 Kouto, Sayo, Hyogo 679-5148, Japan; 2 Japan Synchrotron Radiation Research Institute, 1-1-1, Kouto, Sayo, Hyogo 679-5198, Japan; 3 Institute for Protein Research, Osaka University, 3-2 Yamada-oka, Suita, Osaka 565-0871, Japan

## Abstract

Although the thermodynamics of protein denaturation at temperatures over 100 °C is essential for the rational design of highly stable proteins, it is not understood well because of the associated technical difficulties. We designed certain hydrophobic mutant proteins of CutA1 from *Escherichia coli*, which have denaturation temperatures (*T*_d_) ranging from 101 to 113 °C and show a reversible heat denaturation. Using a hydrophobic mutant as a template, we successfully designed a hyperthermostable mutant protein (*T*_d_ = 137 °C) by substituting six residues with charged ones. Thermodynamic analyses of these mutant proteins indicated that the hydrophobic mutants were stabilized by the accumulation of denaturation enthalpy (Δ*H*) with no entropic gain from hydrophobic solvation around 100 °C, and that the stabilization due to salt bridges resulted from both the increase in Δ*H* from ion-ion interactions and the entropic effect of the electrostatic solvation over 113 °C. This is the first experimental evidence that has successfully overcome the typical technical difficulties.

The tertiary structures of proteins, which are vital for their physiological functions, are related to amino-acid sequences and stabilized by thermodynamic rules. To elucidate the mechanisms of protein folding and protein stabilization, it is critically important to obtain the thermodynamic parameters of protein denaturation as a function of temperature. One difficulty for studying the stabilization mechanism of proteins with denaturation temperatures above 100 °C is that the heat denaturation of proteins is usually irreversible at temperatures higher than 80 °C^1^,^2^,^3^,^4^,^5^,^6^,^7^,^8^, because under these conditions proteins generally aggregate after heat denaturation. Thus, the thermodynamic features of protein stabilization at temperatures above 100 °C are not well understood. One important question in this field is whether the hydrophobic interactions that make the largest contributions to protein stability^9^,^10^,^11^,^12^,^13^ still occur at temperatures above 100 °C^14^,^15^. Next, it is necessary to perform thermodynamic analyses of salt bridges in proteins that have denaturation temperatures above 100 °C, because many proteins from hyperthermophiles seem to be stabilized by an abundance of ion pairs formed by charged residues^8^,^16^,^17^,^18^,^19^,^20^,^21^,^22^,^23^,^24^,^25^,^26^. Thermodynamics of protein denaturation at temperatures over 100 °C is also essentially important for the rational design of hyperthermostable proteins that would be highly useful for industrial and bio-technological processes.

The CutA1 protein from the hyperthermophile *Pyrococcus horikoshii* (*Ph*CutA1) has an unusually high stability, with a denaturation temperature (*T*_d_) of nearly 150 °C at pH 7.0 and an unusually high content of charged residues^26^,^27^. The stabilities and structures of CutA1 proteins from species with various growth temperatures have been also examined, including those of *Thermus thermophilus* (*Tt*CutA1)^28^, *Oryza sativa* (*Os*CutA1)^28^, *Homo sapiens* (brain) (*Hs*CutA1)^29^, and *Escherichia coli* (*Ec*CutA1)^30^. Their *T*_d_ values are also unusually high relative to the growth temperatures of each species: 113.9 °C for *Tt*CutA1, 98.9 °C for *Os*CutA1, 96.2 °C for *Hs*CutA1, and 89.9 °C for *Ec*CutA1. The X-ray crystal structure of *Ph*CutA1 clearly resembles those of other CutA1 proteins. The monomeric structure consists of three α-helices and five β-strands. Three monomers are assembled into a trimer through interactions between the edges of three β-strands (Supplementary Fig. 1). This tightly intertwined interaction contributes to the stabilization of the trimer structures for the CutA1 proteins^26^.

*Ec*CutA1 and its mutants are not heat-reversible^30^. However, other CutA1 proteins, such as *Tt*CutA1 and *Os*CutA1, which have fewer cysteine sulfhydryl (SH) groups, exhibit remarkable heat reversibility^28^. *Ec*CutA1 has three SH groups per subunit. Therefore, we designed an SH-free *Ec*CutA1 mutant, referred to as *Ec*CutA1_0SH (Cys16 → Ala, Cys39 → Ala, Cys79 → Ala), with the aim of achieving high heat reversibility.

In this study, we used the *Ec*CutA1_0SH protein, which has excellent heat reversibility, as a template to design thermostabilized mutants. First, we constructed hydrophobic mutants from *Ec*CutA1_0SH, which were effective in our previous work^30^. Then, to achieve a hyperthermostability comparable to that of *Ph*CutA1, we designed ionic mutants in which charged residues were introduced into a hydrophobic mutant (*Ec*CutA1_0SH_S11V/E61V) by substitution. We describe our use of these *Ec*CutA1 mutants to assess the thermodynamic characteristics of proteins at temperatures over 100 °C, in regard to both hydrophobic and ion–ion interactions.

## Results

### Hydrophobic mutants of *Ec*CutA1 with no SH group

**Figure 1:**
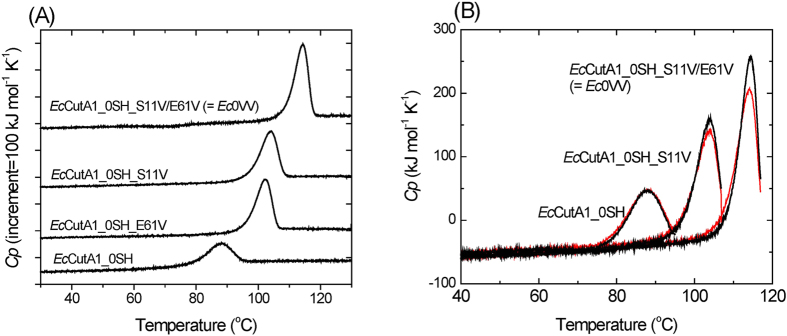
DSC curves of hydrophobic *Ec*CutA1 mutants with no SH group at pH 9.0. (**A**)Typical DSC curves of four mutants: *Ec*CutA1_0SH, *Ec*CutA1_0SH_S11V, *Ec*CutA1_0SH_E61V, and *Ec*CutA1_0SH_S11V/E61V. Scan rates were 60 °C/h. (**B**)Reversibility of heat denaturation of *Ec*CutA1_0SH, *Ec*CutA1_0SH_S11V, and *Ec*CutA1_0SH_S11V/E61V. The red curves of three proteins are the second runs of DSC, just after the cooling step of the first run (the black curves). Scan rates of both curves were 60 °C/h.

We constructed hydrophobic mutants with no SH groups (*Ec*CutA1_0SH_S11V, *Ec*CutA1_0SH_E61V, *Ec*CutA1_0SH_S11V/E61V), which we expected to increase stability^30^. Figure 1A shows typical differential scanning calorimetry (DSC) curves of *Ec*CutA1_0SH and its hydrophobic mutant without SH groups at pH 9.0. As shown in Fig. 1B, the reheating curve (second scan) of *Ec*CutA1_0SH agrees completely with the first scan. The other two proteins also exhibited good reproducibility. These results indicate that the removal of SH groups facilitates excellent reversibility of heat denaturation under these conditions and that we can reliably determine the denaturation enthalpies of these proteins. The denaturation temperature (*T*_d_) of *Ec*CutA1_0SH decreased by 4.3 °C, relative to that (89.9 °C) of *Ec*CutA1 with SH groups, whereas those of *Ec*CutA1_0SH_S11V and *Ec*CutA1_0SH_E61V were 103 and 101 °C, respectively, which were remarkably improved relative to the template. Furthermore, the *T*_d_ of a double mutant, *Ec*CutA1_0SH_S11V/E61V, was 113 °C, which is 28 °C higher than that of the template (Table 1). Hereafter, *Ec*CutA1_0SH_S11V/E61V is abbreviated as *Ec*0VV. These changes in stability due to the hydrophobic mutations were comparable to those observed in mutant proteins with SH groups^30^.

### Ionic mutants of *Ec*CutA1_0SH_S11V/E61V (*Ec*0VV)

**Figure 2:**
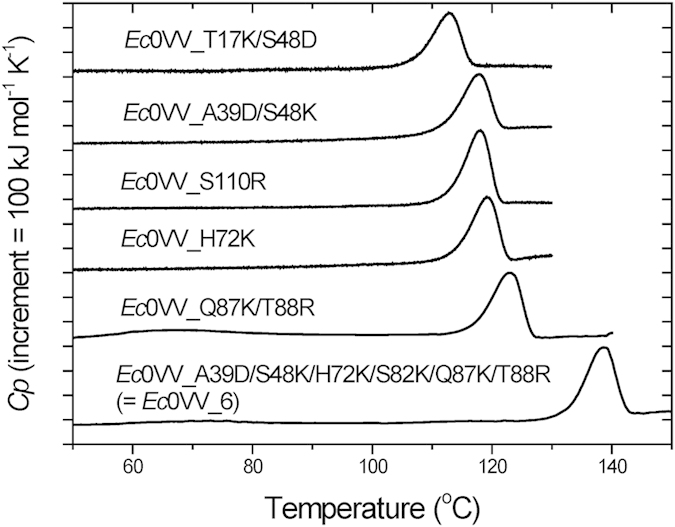
Typical DSC curves of six ionic *Ec*CutA1 mutants from *Ec*0VV at pH 9.0. The six mutants are *Ec*0VV_T17 K/S48D, *Ec*0VV_A39D/S48 K, *Ec*0VV_S110R, *Ec*0VV_H72 K, *Ec*0VV_Q87 K/T88R, and *Ec*0VV_6. Scan rates were 60 °C/h.

To examine the thermodynamic parameters of stabilization by ion–ion interaction at temperatures over 100 °C, we constructed several mutant proteins containing substitutions with charged residues, using *Ec*0VV as a template. Ionic mutants, whose denaturation temperatures are improved and whose DSC curves are suitable for thermodynamic analysis, were selected from our pool of stock mutants (Table 1). Typical DSC curves and reversibility curves are shown in Fig. 2 and Supplementary Fig. 2, respectively. Although the *T*_d_ of a double mutant, *Ec*0VV_T17K/S48D, was lower than that of the template, it was selected because the *T*_d_ was over 100 °C and higher than those of the original single mutants (*Ec*0VV_T17K and *Ec*0VV_S48D) (Table 1). In the case of *Ec*0VV_A39D/S48K/H72K/S82K/Q87K/T88R, which is abbreviated as *Ec*0VV_6, the DSC curve was suitable for analysis (Fig. 2), but the reversibility curve could not be properly obtained due to certain side reactions that occurred at high temperatures. The *T*_d_ of *Ec*0VV_6 was 136.8 ± 0.9 °C, improved by 23.6 °C with the introduction of six charged residues.

In acidic pH, negatively charged residues of a protein should be protonated, leading to a decrease in conformational stability. In the case of CutA1 from *P. horikoshii*, which is stabilized by many ionic interactions, the *T*_d_ of 148.5 °C at pH 7.0 is drastically reduced to 75.6 °C at pH 2.5, whereas the *T*_d_ of CutA1 from *T. thermophilus* changes from 112.8 °C at pH 7.0 to 86.6 °C at pH 2.5^28^. To confirm the stabilization resulted from ionic interactions, the stabilities of ionic mutants were examined under acidic conditions at pH 2–3. The *T*_d_ values of the ionic mutants monotonically decreased as the pH was lowered, reaching a constant minimum at pH 2–2.5 (Supplementary Table 1). We plotted the *T*_d_ shift (*T*_d_ value at pH 9.0 vs. pH 2.0–2.5) versus the *T*_d_ value at pH 9.0 for several ionic mutants (closed circles in Supplementary Fig. 3). Clearly, the *T*_d_ shift became greater as *T*_d_ increased. These results suggest that the electrostatic interactions dominats the thermo-stabilization of the ionic mutant proteins.

### Temperature dependence of denaturation enthalpy at higher temperatures

The denaturation heat capacity (Δ*Cp*) is generally assumed to be constant at temperatures below 80 °C^31^, but it gradually decreases at higher temperatures^14^. Therefore, it is important to elucidate the temperature function of Δ*Cp* at higher temperatures. To this end, we measured the *Cp* values of *Ec*0VV in the native state by DSC at temperatures up to 95 °C (Y2 of Supplementary Fig. 4A). Unfortunately, the temperature dependence of *Cp* values in the denatured state could not be determined experimentally due to the high reversibility of denatured *Ec*0VV. Alternatively, assuming that the heat-capacity contribution of amino-acid groups is additive, the heat capacity of proteins in the denatured state can be calculated from their amino-acid composition^32^. Y1 in Supplementary Fig. 4A also shows the temperature function of the heat capacity of *Ec*0VV in the denatured state, which was estimated from its amino-acid composition using the parameters in Table II of Makhatadze and Privalov^33^. Next, we were able to estimate the temperature function of the denaturation heat capacity (Δ*Cp*) for *Ec*0VV from these native and denatured *Cp* values (Y3 of Supplementary Fig. 4A). The temperature function obtained of Δ*Cp* can be expanded around *T*_d_ as a second-order polynomial:


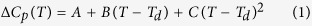


Then, the temperature functions of denaturation enthalpy (Δ*H*) and denaturation entropy (Δ*S*) can be calculated by the following equations (2) and (3), respectively.


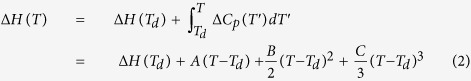



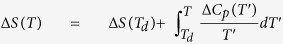



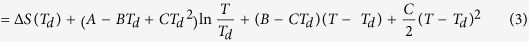


**Figure 3:**
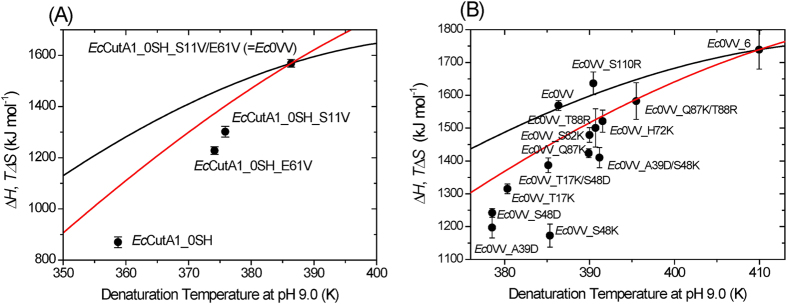
(**A**) Temperature dependence of Δ*H* for hydrophobic *Ec*CutA1 mutants at pH 9.0. Δ*H* values (closed circles) of denaturation for *Ec*CutA1_0SH, *Ec*CutA1_0SH_S11V, *Ec*CutA1_0SH_E61V, and *Ec*0VV come from Table 1. The black curves represent the temperature function of Δ*H* upon denaturation using the temperature function of Δ*Cp* for *Ec*0VV obtained from Y3 of Supplementary Fig. 4A. The red curve represents *T*Δ*S* of *Ec*0VV. In the case of *Ec*0VV, the parameters A, B, and C of Δ*Cp* (in kJ mol^−1^ K^−1^) in equation (1) were calculated to be 7.61029, −0.26614, and −8.4434 × 10^−4^, respectively. (**B**) Temperature dependence of Δ*H* for ionic *Ec*0VV mutants at pH 9.0. Δ*H* values (closed circles) of denaturation for *Ec*CutA1 mutants come from Table 1. Black curves represent the temperature function of Δ*H* upon denaturation, using the temperature function of Δ*Cp* for *Ec*0VV_6 obtained from Y3 of Supplementary Fig. 4B. The red curve represents *T*Δ*S* of *Ec*0VV_6. In the case of *Ec*0VV_6, the parameters A, B, and C of Δ*Cp* (in kJ mol^−1^ K^−1^) in equation (1) were calculated to be 4.22658, −0.29835, and −10.0757 × 10^−4^, respectively.

Figure 3A shows the temperature function of Δ*H* for *Ec*0VV. The Δ*H* values of *Ec*0VV were higher than those at each denaturation temperature of other proteins (*Ec*CutA1_0SH, *Ec*CutA1_0SH_S11V, and *Ec*CutA1_0SH_E61V). If we assume that the temperature function of Δ*Cp* is not largely affected by the constitution of the protein, this observation indicates that stabilization of hydrophobic mutants at residue positions 11 and 61 is mainly caused by enthalpic effects.

The temperature function of Δ*Cp* for *Ec*0VV_6 was also determined using the native *Cp* values (Supplementary Fig. 4B), which were directly measured up to 110 °C. Figure 3B shows the temperature function of Δ*H* for *Ec*0VV_6 and the denaturation enthalpy values at the denaturation temperatures of several ionic *Ec*0VV mutants. This Figure indicates that the Δ*H* value of *Ec*0VV is similar to those of *Ec*0VV_S110R and *Ec*0VV_6 at each denaturation temperature, but remarkably higher than those of other mutants derived from *Ec*0VV by the further addition of charged residues.

**Figure 4:**
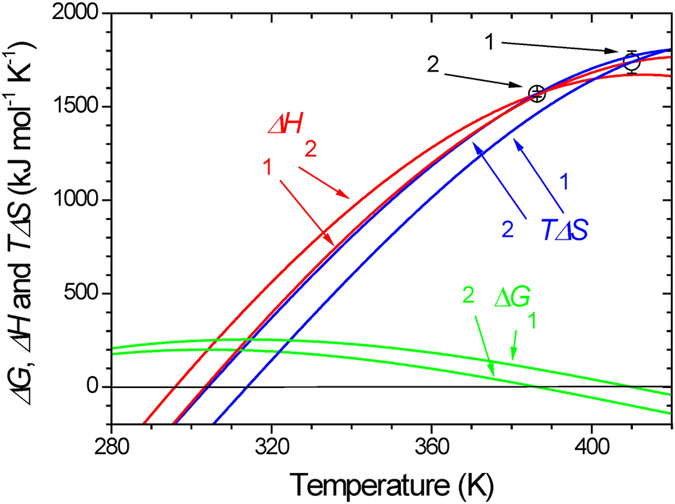
Temperature functions of Δ*G*, Δ*H*, and *T*Δ*S* for *Ec*0VV, and *Ec*0VV_6 between 280 and 420 K. Temperature functions of Δ*H* and Δ*S* were obtained using equations (2) and (3), respectively, in which each temperature function of Δ*Cp* was used for the calculation of *Ec*0VV and *Ec*0VV_6. The green, red, and blue curves represent values of Δ*G*(=Δ*H*–*T*Δ*S*), Δ*H*, and *T*Δ*S*, respectively. Numbers 1 and 2 represent *Ec*0VV_6 and *Ec*0VV, respectively. Open circles with error bars show the Δ*H* value of each protein at the denaturation temperature, as indicated in the figure.

The thermodynamic parameters of denaturation for *Ec*CutA1_0SH mutants at the denaturation temperature (113.2 °C) of *Ec*0VV are listed in Table 2. The Δ*G* values, estimated using the Δ*Cp* temperature function obtained from *Ec*0VV, agreed well with those from *Ec*0VV_6, around the denaturation temperature of *Ec*0VV. Figure 4 also shows the temperature functions of Δ*G*, Δ*H*, and *T*Δ*S* for *Ec*0VV and *Ec*0VV_6 over a larger temperature range (between 280 and 420 K), indicating that Δ*G* values of *Ec*0VV_6 are positive over a broad range of temperatures.

## Discussion

Forty years ago, Privalov *et al.*^14^,^31^ developed highly qualified adiabatic differential micro-calorimeters for determining the thermodynamic parameters of protein denaturation. They reported that specific characters of amino-acid residues disappear during protein unfolding near 110 °C, and that all the observed entropy originates from the increase in conformational freedom of the polypeptide upon unfolding, because the specific Δ*H* and Δ*S* of unfolding for several proteins intersect at a single point near 110 °C. On the other hand, the thermodynamics of transfer of hydrocarbons to water provides a model for the temperature dependence of the hydrophobic interaction in protein folding. Baldwin^15^ examined the solution thermodynamics of several liquid hydrocarbons in water. He found that the extrapolated temperatures at which the transfer Δ*S* reaches zero, around 112.8 °C, were similar for six hydrocarbons. The extrapolated temperature of the transfer Δ*H* is 22.0 °C. This means that at 113 °C, the hydrophobic interaction changes from being entropy-driven at 22 °C to being enthalpy-driven at 113 °C, and the contribution of water to the entropy of protein unfolding (hydrophobic hydration) is removed^15^. Regarding these estimations, the heat capacity change (Δ*Cp*) is assumed to be constant against temperature. Later, Makhatadze and Privalov^33^,^34^ reported that the temperature at which Δ*S* is zero approaches 145 °C when the decreasing nature of Δ*Cp* against temperature is taken into account, because the Δ*Cp* value of hydrocarbon hydration decreases with increasing temperature.

In this study, the heat capacities of the native states of *Ec*0VV and *Ec*0VV_6 could be directly measured by DSC up to 95 and 110 °C, respectively, and the temperature functions of their Δ*Cp* values were estimated as shown in Supplementary Fig. 4A and Supplementary Fig. 4B, respectively.

### Hydrophobic effects strongly contribute to Δ*H* at temperatures around 100 °C

The temperature function of Δ*H* of *Ec*0VV is depicted in Fig. 3A. Using the same temperature function of Δ*Cp*, the Δ*H* values of *Ec*0VV, *Ec*CutA1_0SH_S11V, *Ec*CutA1_0SH_E61V, and *Ec*CutA1_0SH were estimated to be 1569, 1396, 1340, and 1175 kJ/mol at 113.2 °C, respectively (Table 2). The increase in Δ*H* (394 kJ/mol) of *Ec*0VV (*Ec*CutA1_0SH_S11V/E61V) agrees well with the sum (386 kJ/mol) of the increases in Δ*H* of *Ec*CutA1_0SH_S11V (221 kJ/mol) and *Ec*CutA1_0SH_E61V (165 kJ/mol). On the other hand, the *T*Δ*S* values of *Ec*0VV (the red curve in Fig. 3A) were larger than the Δ*H* value (=*T*Δ*S* at *T*_d_) at each *T*_d_ of *Ec*CutA1_0SH_S11V, *Ec*CutA1_0SH_E61V, and *Ec*CutA1_0SH, indicating that *Ec*0VV is entropically unfavorable compared with other proteins. That is, *Ec*0VV, which contains hydrophobic substitutions for hydrophilic residues in the interior of a molecule, is mainly stabilized by the enthalpic gain upon substitutions, but is partly destabilized by the entropic loss, probably due to the disruption of the hydrophilic solvation in the denatured state upon substitutions. These results at high temperatures around 100 °C are contrary to the well-accepted belief that the entropic gain from hydrophobic solvation can account for the stabilization effect of hydrophobic substitutions at lower temperatures^15^. Whereas, the estimations from the hydration of amino acids^34^ are generally consistent with our results. This is the first experimental evidence pertaining to the hydrophobic effects on protein stability, which is obtained by direct measurement at temperatures around 100 °C.

### *Ec*0VV_6 substituted with six charged residues is stabilized by both enthalpic and entropic effects around 137 °C

Below 100 °C, the ion–ion interaction (salt bridge) is driven entirely by entropic effects due to the release of water strongly bound to the ions of charged residues^35^,^36^,^37^. The proteins substituted with single charged residues, *Ec*0VV_H72K, *Ec*0VV_S82K, *Ec*0VV_Q87K, and *Ec*0VV_T88R, are stabilized by electrostatic interactions (Supplementary Fig. 3). The Δ*H* values of all of these proteins were drastically decreased relative to Δ*H* at each corresponding temperature on the temperature function of *Ec*0VV_6 (Fig. 3B). Because the changes in Δ*H* upon these mutations are unfavorable for folding, the observed improvements in stability were caused by entropic effects due to the release of water at the charged residues that we introduced (electrostatic solvation). The thermodynamic analyses also clearly confirmed stabilization resulted from entropic effects (Table 2). The other single mutants, *Ec*0VV_A39D and *Ec*0VV_S48 K, whose mutation sites were located in the interior of the molecule (Table 3), demonstrated drastically decreased *T*_d_ and Δ*H* values. However, a double mutant containing both of these single mutants, *Ec*0VV_A39D/S48 K, in which Asp39 forms a strong salt bridge with Lys48, was stabilized by an increase in Δ*H* relative to the single mutants (Fig. 3B), probably due to Coulomb’s force resulting from salt bridge formation. A similar result was obtained in the other double mutant, *Ec*0VV_T17 K/S48D (Fig. 3B). The double mutant *Ec*0VV_Q87 K/T88R was also stabilized by an increase in Δ*H* relative to *Ec*0VV_Q87 K and *Ec*0VV_T88R. Because this double mutant does not have additional salt bridges, two individual thermo-stabilizations might work synergistically at around 120 °C to promote the desolvation of the ionic residues introduced, thereby reducing both the enthalpic loss and the entropic gain that are mutually attributed to the electrostatic solvation. In addition, the increase in Δ*H* in this double-ion mutant might have been caused by a hydrophobic interaction due to the alkyl groups of Lys87 and Arg88.

*Ec*0VV_6, with six additional charged residues, was stabilized by an increase in the Δ*H* value relative to each of the six original ionic mutants (Fig. 3B). The increase in Δ*H* might indicate that hydrophobic effects due to the alkyl groups of Lys or Arg and Coulomb’s force still function effectively at these high temperatures. According to Coulomb’s law, the strength of an electrical interaction is inversely proportional to the dielectric constant. The dielectric constant of water drops from 80 at 0 °C to 55 at 100 °C^38^. Furthermore, Elcock^39^ found that increasing temperature decreases the electrostatic desolvation penalty incurred in forming a salt bridge, leading to an increase in salt bridge stabilization from his continuum solvation model^40^. The increase in Δ*H* of *Ec*0VV_6 might result mainly from the high degree of desolvation of the ionic residues in the denatured state at around 137 °C, in addition to the other effects described above.

The temperature dependence of Δ*H* for *Ec*0VV_6 has an intersection near *T*_d_ (113 °C) of *Ec*0VV, as shown in Fig. 4, suggesting that the contribution of Δ*H* to the stability of *Ec*0VV_6 with additional 6 charged residues becomes favorable in the temperature region above 113 °C, compared with those of *Ec*0VV. Furthermore, Fig. 4 shows that the increase in Δ*G* of *Ec*0VV_6 results largely from the decrease in Δ*S* of *Ec*0VV_6 when compared with the template *Ec*0VV at temperatures below 113 °C. That is, the stabilization due to ionic mutations results mainly from both the enthalpic gain from ion-ion interactions in the native state and the entropic gain from the water release of ionic residues in the denatured state at temperatures over 113 °C.

A mutant, *Ec*0VV_S110R, whose substitution position is located in the C-terminus of α-helix and almost buried (Table 3), was stabilized by an increase in Δ*H* (Fig. 3B). In this case, due to local conformations, the decrease in Δ*H* due to water release might be suppressed by the effects of other stabilizing factors.

## Conclusion

The rational design of hyper-thermostable proteins may be possible through the introduction of multiple salt bridges at high temperatures over 113 °C that can be reached through a preceding stabilization by hydrophobic substitutions.

## Matrials and Methods

### Mutagenesis, expression, and purification of CutA1 mutants from *E. coli*

The mutagenesis, expression, and purification of CutA1 mutants from *E. coli* were performed as described^30^ with minor modifications. The homogeneity and identity of the purified samples were assessed using SDS–PAGE. The protein concentration was estimated from the absorbance at 280 nm, assuming E^1cm^_1%_ = 14.96, based on the number of aromatic amino acids^41^.

### Differential scanning calorimetry (DSC) experiments

To measure the changes in stability due to mutations, DSC was performed using a scan rate of 60 °C/h on a VP-capillary DSC platform (Microcal, USA) for temperatures up to 130 °C at pressures below 60 psi, or a Nano-DSC 6300Y microcalorimeter (TA Instruments, USA) for higher temperatures up to 160 °C at a pressure of 88 psi. Protein concentrations were around 0.6 mg/ml in a 50 mM glycine buffer at pH 9.0 containing 2 mM EDTA or a 50 mM glycine buffer at pH 2.0–3.5. All samples were dialyzed against the buffers overnight at 4 °C and then filtered through a membrane with 0.22-μm pores. The denaturation temperature (*T*_d_) is the temperature at which the area of the denaturation enthalpy (Δ*H*) is 0.5. The *T*_d_ and Δ*H* values in this study represent the averages for at least six experiments.

To measure the heat capacity of mutant proteins in their native states, the protein concentrations were adjusted to around 2.0 mg/ml in a 50 mM glycine buffer at pH 9.0. Two different scan rates, 60 and 200 °C/h, were used. Each experiment comprised six cycles of reheating to the pre-denaturation temperatures: 95 °C and 110 °C for *Ec*0VV and *Ec*0VV_6, respectively. The partial specific volumes for the calculation of heat capacity were estimated from the amino-acid composition of each mutant protein^42^.

## Additional Information

**How to cite this article**: Matsuura, Y. *et al.* Thermodynamics of protein denaturation at temperatures over 100 °C: CutA1 mutant proteins substituted with hydrophobic and charged residues. *Sci. Rep.* **5**, 15545; doi: 10.1038/srep15545 (2015).

## Supplementary Material

Supplementary Information

## Figures and Tables

**Table 1:** Denaturation enthalpy of examined *Ec*CutA1_0SH mutants at denaturation temperatures.

	*T*_d_( °C)*			Δ*H* (kJ mol^−1^)*		
*Ec*CutA1_0SH	85.6	±	0.3	870	±	21
*Ec*CutA1_0SH_S11V	102.7	±	0.3	1302	±	21
*Ec*CutA1_0SH_E61V	101.0	±	0.2	1228	±	15
*Ec*0VV**	113.2	±	0.2	1569	±	15
*Ec*0VV_T17K	107.2	±	0.3	1315	±	15
*Ec*0VV_S48D	105.4	±	0.2	1242	±	14
*Ec*0VV_T17K/S48D	112.0	±	0.1	1387	±	22
*Ec*0VV_A39D	105.4	±	0.5	1197	±	31
*Ec*0VV_S48 K	112.2	±	0.9	1173	±	35
*Ec*0VV_A39D/S48 K	118.3	±	0.7	1410	±	33
*Ec*0VV_H72 K	118.4	±	0.4	1521	±	34
*Ec*0VV_S82 K	116.9	±	0.5	1479	±	22
*Ec*0VV_S82R	117.1	±	0.5	1446	±	24
*Ec*0VV_T88R	117.6	±	0.6	1501	±	58
*Ec*0VV_Q87 K	116.8	±	0.5	1424	±	13
*Ec*0VV_Q87K/T88R	122.4	±	0.6	1582	±	56
*Ec*0VV_S110R	117.3	±	0.4	1637	±	35
*Ec*0VV_6***	136.8	±	0.9	1739	±	59

^*^Average value and its standard deviation of at least 6 data.

^**^Ec0VV represents *Ec*CutA1_0SH_S11V/E61V mutant.

^***^Ec0VV_6 represents *Ec*0VV_A39D/S48K/H72 K/S82 K/Q87 K/T88R mutant.

**Table 2:** Thermodynamic parameters of denaturation for *Ec*CutA1_0SH mutants at the denaturation temperature (113.2 ^o^C) of *Ec*0VV.

	Δ*H*		*T*Δ*S*		Δ*G (=*ΔΔ*G)*		ΔΔ*H*		*T*ΔΔ*S*	
	a*	b*	a	b	a	b	a	b	a	b
*Ec*CutA1_0SH	1175	1254	1255	1336	−80	−82	−394	−315	−314	−233
*Ec*CutA1_0SH_S11V	1396	1440	1434	1478	−38	−38	−173	−129	−135	−91
*Ec*CutA1_0SH_E61V	1340	1377	1382	1419	−42	−42	−229	−192	−187	−150
*Ec*0VV	1569	1569	1569	1569	0	0	0	0	0	0
*Ec*0VV_A39D	1265	1289	1290	1315	−26	−26	−304	−280	−279	−254
*Ec*0VV_S48 K	1181	1184	1184	1187	−3	−3	−388	−385	−385	−382
*Ec*0VV_A39D/S48 K	1375	1358	1356	1340	18	18	−194	−211	−213	−229
*Ec*0VV_H72 K	1485	1469	1465	1449	20	20	−84	−100	−104	−120
*Ec*0VV_S82 K	1453	1442	1439	1428	14	14	−116	−127	−130	−141
*Ec*0VV_T88R	1470	1456	1453	1440	17	17	−99	−113	−116	−129
*Ec*0VV_Q87 K	1398	1387	1385	1374	13	13	−171	−182	−184	−195
*Ec*0VV_Q87 K/T88R	1524	1495	1488	1459	36	36	−45	−74	−81	−110
*Ec*0VV_S110R	1608	1595	1590	1578	17	17	39	26	21	9
*Ec*0VV_6	1638	1560	1540	1465	98	96	69	−9	−29	−104

The unit is kJ mol^−1^.

^*^a and b represent the calculated results using the temperature function of Δ*Cp* obtained from *Ec*0VV and *Ec*0VV_6, respectively.

**Table 3:** Burial rates of target residues of ***Ec*** 0VV mutants*.

Mutants	Residues	All atoms			Charged atoms			location
*Ec*0VV_6	Asp39	100	±	1	99	±	2	beta 2
	Lys48	96	±	3	93	±	6	beta 2
	Lys72	80	±	5	55	±	18	alpha 2, N terminal
	Lys82	10	±	6	13	±	8	alpha 2, C terminal
	Lys87	10	±	14	−2	±	21	loop
	Arg88	55	±	20	40	±	34	loop
	Val11	98	±	1				beta 1
	Val61	97	±	3				beta 3
*Ec*0VV_S110R	Arg110	84	±	10	66	±	20	alpah 3, C-terminal

^*^The burial rates (%) were estimated from the average of ASA values for nine structures during 40 ns MD at 300 K (in preparation).
