# Supplementary material for: Thermodynamics of protein denaturation at temperatures over 100 °C: CutA1 mutant proteins substituted with hydrophobic and charged residues
Source: Sci Rep. 2015 Oct 26;5:15545. doi: 10.1038/srep15545 (PMC4620440; doi:10.1038/srep15545)
Supplement: Supplementary Information [file srep15545-s1.pdf]

Supplementary Information

Thermodynamics of protein denaturation at temperatures over 100°C: CutA1 mutant proteins substituted with hydrophobic and charged residues

Yoshinori Matsuura[1], Michiyo Takehira[1], Yasumasa Joti[2], Kyoko Ogasahara[3], Tomoyuki Tanaka[1], Naoko Ono[1], Naoki Kunishima[1], and Katsuhide Yutani[1,*]

[1]RIKEN SPring-8 Center, 1-1-1 Kouto, Sayo, Hyogo 679-5148, Japan

[2]Japan Synchrotron Radiation Research Institute, 1-1-1, Kouto, Sayo, Hyogo 679-5198, Japan

[3]Institute for Protein Research, Osaka University, 3-2 Yamada-oka, Suita, Osaka 565-0871, Japan

Supplementary Table 1. Denaturatioin temperatures of *Ec* 0VV mutants in the acidic region

	pH						
Mutants	3.0	2.9	2.7	2.5	2.25	2.0	2.5-2.0*
Ec 0VV	85.9	85.0	82.6	80.6	80.4	81.0	80.7
Ec 0VV_A39D/S48K	85.6	82.0	77.3	75.1	73.6	74.9	74.5
Ec 0VV_H72K	88.7	87.2	84.5	83.4	82.5	83.2	83.0
Ec 0VV_S82K	84.3	81.8	78.6	76.7	76.9	76.7	76.7
Ec 0VV_S82R	83.8	82.2	79.2	78.1	78.6	78.0	78.2
Ec 0VV_Q87K	83.2	81.1	78.2	76.6	76.7	76.9	76.7
Ec 0VV_T88R	83.2	80.8	77.8	76.5	76.8	76.4	76.6
Ec 0VV_S110R	84.2	82.4	80.1	78.5	78.3	79.0	78.6
Ec 0VV_6	85.3	80.8	73.3	70.1	69.6	68.5	69.4

Each data represents average of two data.

The unit of data is °C.

*Average value of pH 2.5, 2.25, and 2.0.

Supplementary Figure 1

The trimer crystal structure of *Ec*CutA1_0SH (PDB ID 4Y65). Different colors represent different chains. α and β represent α helix and β strand, respectively. Three N terminal residues of B subunit (cyan) and eight N terminal residues of C subunit (magenta) are missing in the crystal structure.

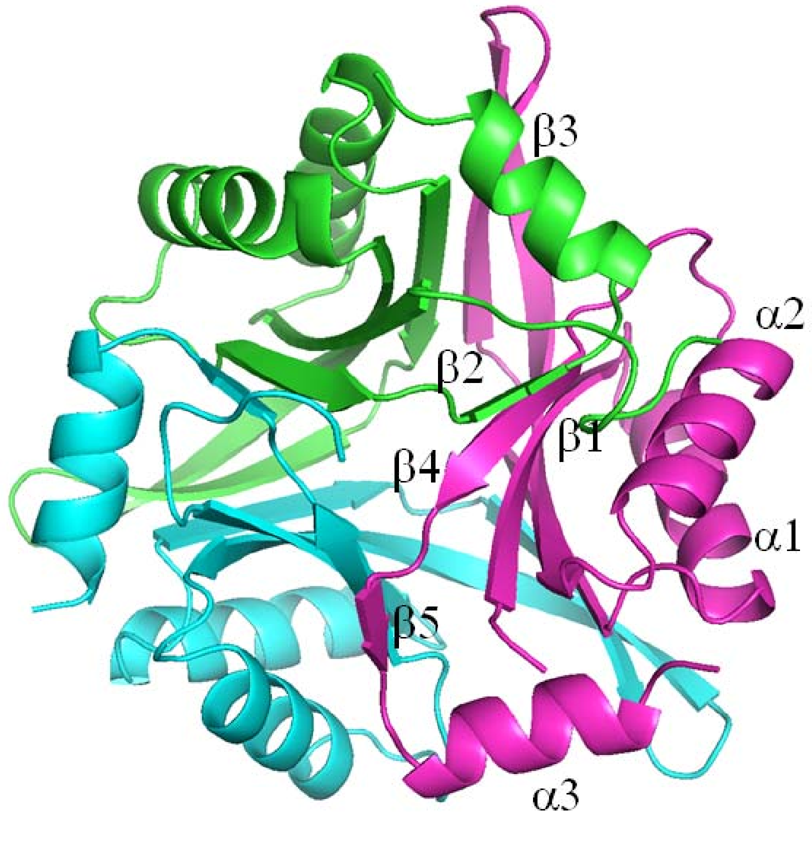

Supplementary Figure 2

Reversibility of the DSC curves of ionic mutants from *Ec*0VV at pH 9.0. Red curves are the second runs of DSC just after cooling of the first run (black curves). Scan rates of both curves were 60ºC/h

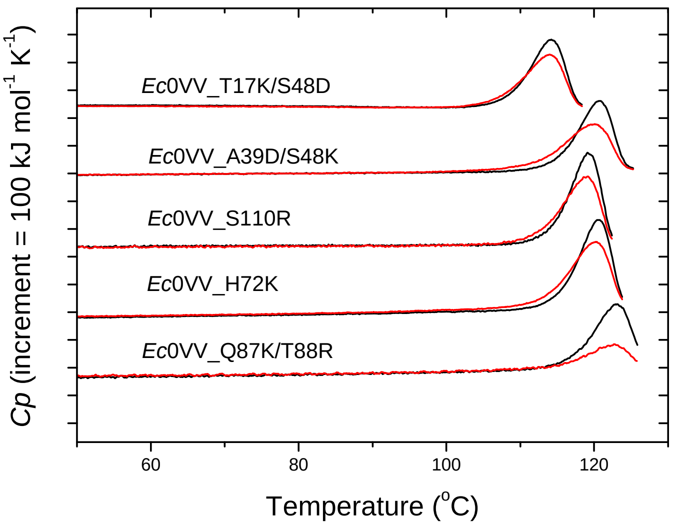

Supplementary Figure 3

Comparison between T_d values of ionic mutants of *Ec*0VV at pH 9.0 and pH 2.0–2.5.

Open circles represent average T_d values at pH 2.0, 2.25, and 2.5 for ionic mutants of *Ec*0VV (Table S1). Closed circles represent shifted temperatures, which are the differences between the T_d values at pH 9.0 (Table 1) and the average T_d values at pH 2.0–2.5 for the ionic mutants of *Ec*0VV (Supplementary Table 1). Lines A and B represent linear regressions for open and closed circles, respectively. Numbers 1–9 represent mutant proteins of *Ec*0VV, *Ec*0VV_6, *Ec*0VV_A39D/S48K, *Ec*0VV_H72K, *Ec*0VV_S82K, *Ec*0VV_S110R, *Ec*0VV_S82R, *Ec*0VV_Q87K, and *Ec*0VV_T88R.

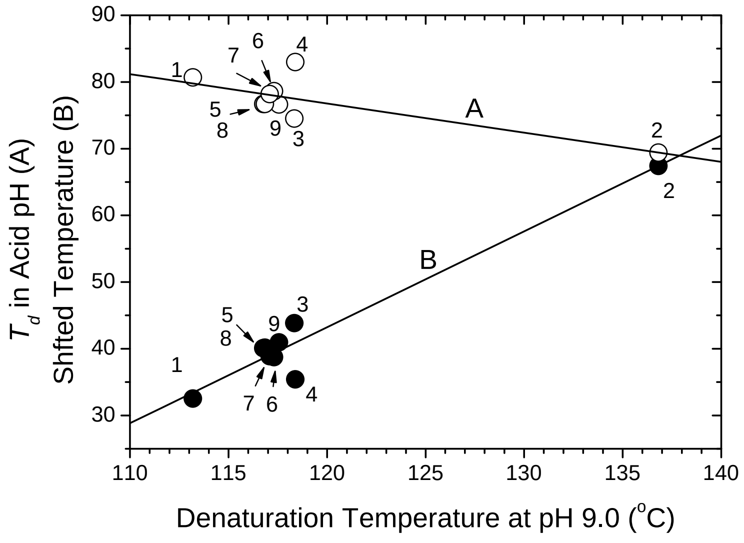

Supplementary Figure 4

(A) Temperature dependence of *Cp* for *Ec*0VV in the native and denatured states. Y1 represents the temperature dependence of *Cp* ($Jg^{-1}K^{-1}$) for *Ec*0VV in the denatured state. Black closed circles in Y1 represent the heat capacity of *Ec*0VV in the denatured state, estimated from the amino-acid composition using the parameters in Table II of Makhatadze and Privalov[33]. The Y1 curve is the result of fitting a secondary expression to the data. Curve Y1 = $-1.01674 + 1.753 \times 10^{-2}T - 2.282 \times 10^{-5}T^2$, where T is the temperature in Kelvin. Y2 represents the heat capacity in the native state; small circles represent experimental data. Each experiment comprised the six times cycles of reheating to the pre-denaturation temperature. The data points (small circles) show all data in 3 time experiments of the liner regression obtained from each experiment. The liner line in Y2 is liner regression of all data shown in the figure. Line Y2 = $-0.56817 + 0.709 \times 10^{-2}T$. Y3 represents the temperature function of denaturation heat capacity, ΔCp, between the native and denatured states, i.e., Y3 = Y1 – Y2. Thus, Y3 = $-0.44857 + 1.044 \times 10^{-2}T - 2.282 \times 10^{-5}T^2$.

(B) Temperature dependence of *Cp* for *Ec*0VV_6 in the native and denatured states. Y1 represents the temperature dependence of *Cp* ($Jg^{-1}K^{-1}$) for *Ec*0VV_6 in the denatured state. Black closed circles and a curve in Y1 show the heat capacity of *Ec*0VV_6 in the denatured state, estimated as shown in the legend of Supplementary Fig. 4A. Curve Y1 = $-0.98373 + 1.735x10^{-2}T - 2.25855x10^{-5}T^2$, where T is a temperature in Kelvin. Y2 represents the heat capacity in the native state, where small circles are experimental data. Each experiment comprised the six times cycles of reheating to the pre-denaturation temperature. The liner line in Y2 is liner

regression of all data shown in the figure. Line $Y2 = -0.38148 + 0.645 \times 10^{-2}T$. Y3 represents ΔCp, between the native and denatured states, i.e., Y3 = Y1 – Y2. Thus, $Y3 = -0.60225 + 1.09 \times 10^{-2}T - 2.2586 \times 10^{-5}T^2$.

Fig. 4A

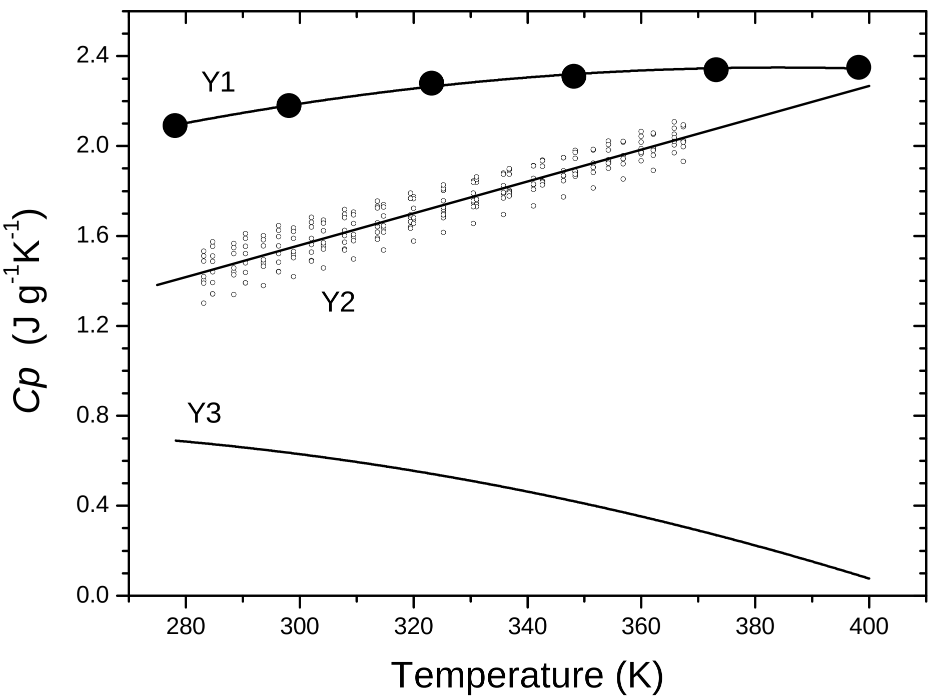

Fig. 4B

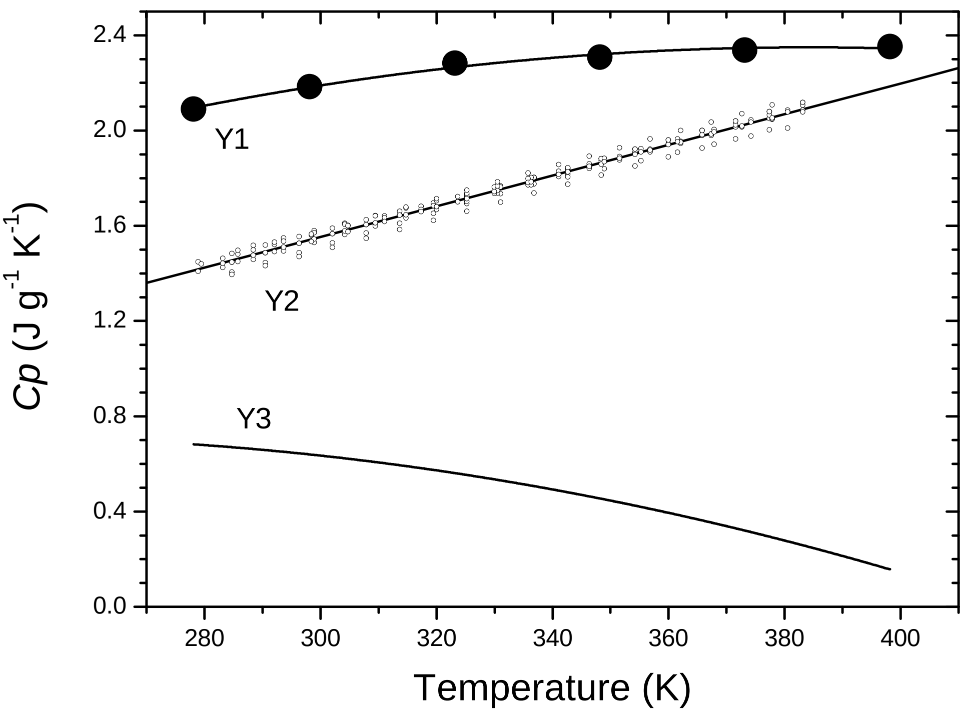
2.4
2.0
1.6
1.2
0.8
0.4
0.0
280
300
320
340
360
380
400
Cp (J g-1 K-1)
Temperature (K)
Y1
Y2
Y3
